# Crosstalk between cohesins and axis proteins determines meiotic chromosome architecture in *Sordaria macrospora*

**DOI:** 10.1371/journal.pgen.1012001

**Published:** 2025-12-23

**Authors:** Kenza Yefsah, Marwan Habbi, Karine Budin, Henri-Marc Bourbon, Denise Zickler, Eric Espagne, Stéphanie Boisnard

**Affiliations:** 1 Université Paris-Saclay, Commissariat à l’Énergie Atomique et aux Énergies Alternatives (CEA), Centre National de la Recherche Scientifique (CNRS), Institute for Integrative Biology of the Cell (I2BC), Gif-sur-Yvette, France; 2 Centre de Biologie Intégrative, Molecular, Cellular Developmental Biology Unit, Université Fédérale de Toulouse, Toulouse, France; University of Georgia, UNITED STATES OF AMERICA

## Abstract

Faithful chromosome segregation during meiosis requires the coordinated action of cohesin complexes and chromosome axis proteins. How these factors interact and communicate along chromosome axes, especially during meiotic prophase I, remains however, only partially understood. We therefore investigated the functional interplay between the cohesin components and regulators (Rad21, Rec8, Wapl, Sororin, Spo76/Pds5) and two meiosis-specific axis proteins Red1 and Hop1. Analysis of multiple combinations of their corresponding null mutants and of their genetic-epistasis interactions in the fungus *Sordaria macrospora* revealed a hierarchical regulatory network for their recruitment and releasing. This work uncovers an unexpected role of axis proteins Red1 and Hop1, that together with Sororin, provide stage-specific protection of Spo76/Pds5 against Wapl-mediated release. Furthermore, we identify that Spo76/Pds5 is the main target of Wapl and acts as a central guardian of kleisin stability against Slx8/STUbL-dependent proteasomal degradation. Together, our findings show that dynamic crosstalk between axis proteins and cohesins is crucial to preserve axis integrity and to ensure accurate meiotic progression.

## Author summary

Meiosis is the process that produces gametes with the correct number of chromosomes. For this to happen, chromosomes must be carefully organized and held together in a highly controlled manner. Cohesin complexes, which form ring-like structures around DNA, shape chromosome architecture and hold sister chromatids together, and their stability is controlled by proteins that either maintain or release them.

During meiosis, cohesins work together with axis proteins such as Red1 and Hop1, but how these components coordinate their functions has remained unclear. In the fungus *Sordaria*, chromosome axes form early and continuously, providing an ideal system for understanding how cohesins and axis proteins interact during early meiosis. By studying mutants lacking key regulatory proteins, we uncovered how different factors protect cohesin stability at specific stages and prevent cohesins from being removed too early. We also discovered that the cohesin regulator Spo76/Pds5 plays a central role in cohesins stability. Together, these findings uncover a regulatory network in which axis proteins and cohesins support each other to maintain chromosome-axis integrity and to ensure accurate meiotic progression.

## Introduction

The majority of cellular processes rely on a complex three-dimensional chromatin organization, involving nucleosomes and post-translational modifications of histones, which represent the primary level of chromatin organization [1–3]. Beyond this fundamental organization, chromatin undergoes dynamic remodeling throughout the cell cycle, orchestrated by Structural Maintenance of Chromosomes (SMC) complexes such as cohesin complexes, condensins and the Smc5/6 complex [4].

SMC family complexes are evolutionarily conserved across organisms. Their ring-like structure allows them to entrap DNA molecules, which, in turn, generates a higher-order chromatin organization whose regulation and dynamics ensure a wide range of essential cellular functions [5–7].

The cohesin complex, a key member of the SMC family, consists of two ATPases, Smc1 and Smc3, which form an antiparallel coiled-coil dimer through interactions at their hinge domains. The dimer is bridged by the kleisin subunit Scc1/Rad21/Mcd1, which closes the ring and recruits regulatory proteins such as Scc3, essential for cohesin activity [8–10]. Additional regulators include the loader complex Scc2/NIPBL-Scc4 [11], and the accessory factors Spo76/Pds5 [12], Wapl/Rad61 [13], and Sororin [14,15]. Specific interactions among these cohesin components underlie their distinct roles in chromatin organization and sister chromatid cohesion.

Before DNA replication, cohesin complexes associate dynamically with chromosome axes and this association is under the control of the releasing activity of Wapl [16]. Following replication, association of cohesin with chromatin must be stabilized in a “cohesive state” to ensure sister chromatid cohesion. This cohesive state, is achieved by inhibition of Wapl activity. In mammals, Smc3 acetylation by Eco1-family acetyltransferases (Esco1/2) promotes Spo76/Pds5-Sororin interaction, thereby antagonizing Wapl [14,17–21]. In budding yeast, which lacks a Sororin ortholog, Smc3 acetylation, protected from Hos1 deacetylation by Spo76/Pds5, is sufficient to inhibit Wapl activity [22–24]. Moreover, Spo76/Pds5 also safeguards cohesins from SUMO-ubiquitin-proteasome-mediated degradation, and is itself SUMOylated, modulating its protective activity [25–27].

Beyond their essential role in sister chromatid cohesion, cohesin complexes also regulate chromosome architecture by driving loop formation through a loop extrusion model [13,28].

Meiosis introduces additional layers of complexity compared to mitosis. Meiotic prophase I is markedly prolonged, particularly in mammals and plants [29], requiring sustained cohesin protection to prevent premature sister-chromatid separation. Cohesin removal occurs in two steps, with chromosome-arm cohesion released at anaphase I to resolve chiasmata and thus separate homologs, whereas centromeric cohesion is preserved until anaphase II (review in [30,31]). Thus, meiotic sister-chromatid cohesion requires stringent spatial and temporal regulation. This regulation is orchestrated by the same cohesin regulators (Scc2/Scc4, Spo76/Pds5, Wapl/Rad61, and Sororin) as during mitosis [12,32–37].

In addition, cohesin complexes are composed by some meiosis-specific components during the meiotic process. While the meiosis-specific kleisin Rec8 is conserved in all organisms studied so far, specific cohesin complexes exist in mammals which involve the kleisin, Rad21L, as well as meiotic variants of Smc1 and Scc3 proteins, i.e.,: Smc1β and STAG3, respectively (review in [2,38]). Consequently, distinct cohesin complex populations coexist and likely contribute differentially to chromatin axis-loop formation and sister-chromatid cohesion during meiosis.

During meiotic prophase I, the chromosome axis also includes structural proteins such as Red1 (homologous to SYCP2/SYCP3 in mammals and ASY3/ASY4 in plants) and the HORMA-domain protein Hop1 (HORMAD1/2 in mammals, ASY1/PAIR2 in plants), which are essential for homolog pairing, synapsis, and recombination [2,3,39,40]. This raises the question of how cohesin complexes interact with these axis-associated proteins to regulate meiotic progression during prophase I.

Using the model organism *Sordaria macrospora*, we explored the functional and epistatic interactions between two chromosomal axis proteins (Hop1 and Red1) and five cohesin components (Rec8, Scc1/Rad21/Mcd1, Spo76/Pds5, Wapl and Sororin). *Sordaria* provides a particularly attractive experimental system for such analysis because, contrary to mammals and budding yeast, chromosome axes emerge in a cytologically detectable form concomitant with DNA replication and occur as continuous units from mid-leptotene on. This allows a careful analysis of the hierarchical regulation of the seven proteins and their collective impact on chromosome organization and dynamics during meiotic prophase I.

The present study includes identification of *Sordaria* Wapl, Sororin and Red1 as novel components of this fungus chromosome axes. The three proteins localize along axes from leptotene to end pachytene similarly to previously characterized axis-associated proteins Spo76/Pds5, Rec8 and Hop1 [12,41,42]. Through analysis of multiple combination of their corresponding null mutants and of their epistasis, we provide several key insights concerning their relationship. These findings contribute to a deeper understanding of the molecular architecture and regulatory hierarchy governing meiotic chromosome axis formation and function.

## Results

### Identification of *Sordaria macrospora RED1*, *WAPL* and *SORORIN*

***RED1*.** Combined phylogeny-oriented structural homology searches using Foldseek, HMMsearch, and PSI-BLAST, together with multiple sequence alignments across fungal genomes, identified a single *RED1* gene (SMAC4_01425) in *Sordaria.* The predicted Red1 protein (839-amino acids) contains a 244-residue C-terminal region (aa 583–769) analysed by DeepCoil2 to adopt a coiled-coil (CC) structure (S1A Fig). Multiple sequence alignments of the N-terminal residues from fungal orthologs (S1A Fig), combined with AlphaFold3 (AF3) structural modeling, revealed two conserved domains: (i) a 30-residue N-terminal motif likely mediating Hop1 interaction (ipTM: 0.71; pTM: 0.83; S1A, S1B and S1C Fig), and (ii) a 21-residue motif predicted to mediate Mek1 interaction (ipTM: 0.54; pTM: 0.62; S1A, S1B and S1D Fig). As previously reported in *S. cerevisiae* [43], AF3 modeling predicts that the *Sordaria* Red1 CC region mediates self-association (S1B and S1E Fig), consistent with its structural role in axis organization. In addition, AF3 modeling predicts interactions of Red1 with Rec114 and Zip4 consistent with functional roles in meiotic chromosome axis assembly [44,45] (S1B, S1F and S1G Fig). Together, our phylogenetic and structural modeling analyses strongly support that SMAC4_01425 encodes *a bona fide RED1* homolog.

***WAPL*** was identified by PSI-BLAST using the *Schizosaccharomyces pombe* Wapl protein sequence as a query. The S*ordaria* genome contains a single *WAPL* gene (SMAC4_07015). The predicted Wapl protein (852 amino acids) displays the domain organization shown in S2A Fig. It contains a conserved C-terminal domain (aa 380–726; S2A Fig), that defines the Wapl family and is predicted to be predominantly α-helical [13]. In contrast, the highly divergent N-terminal region (variable in both length and composition) is thought to mediate interaction with Pds5 [46]. AlphaFold2 (AF2) modeling (S2B and S2C Fig showing prediction confidence values) indicate that this N-terminal region is intrinsically disordered, as previously proposed for other Wapl orthologs. AF2 modeling of the C-terminal region (aa 380–852), combined with structural similarity searches against experimentally determined atomic structures in the Protein Data Bank, revealed that the *Sordaria* Wapl domain shares highest structural homology with the *Ashbya gossypii* Wapl domain (PDB: 3zik, Chain B; (S2D Fig). Structurally, the *Sordaria* Wapl domain adopts a fold of 25 consecutive α-helices, 15 of which align with the 21 α-helices present in *A. gossypii* ortholog (Z-score: 5.084). The Wapl domain can be subdivided into three regions, with the central region comprising six HEAT repeats [47].

***SORORIN***. *Sordaria* Sororin (SMAC4_08091) is a 701-amino acids protein, which, like all members of this family, is largely unstructured and poorly conserved except for the Sororin boxes [36]. Orthologs are found in human, insects, plants, and fungi except for *S. cerevisiae* which lacks a potential ortholog [36]. Sororin is characterized by a very short C-terminal domain (Sororin boxes), consisting of a polar linker (K/R-rich domain which varies in size between 10 and 20 amino acids) and a conserved motif predicted to form two α-helices and a β-strand [21]. Vertebrate Sororin and Wapl proteins interact with Pds5 through their YSR and FGF motifs [21,46]. The putative FGF and KEN box motifs, are not present in the *Sordaria* protein. In vertebrates, the KEN box mediates Sororin degradation during mitotic exit, ensuring timely cohesin removal. Absence of this motif in *Sordaria* suggests an alternative degradation mechanism, potentially involving ubiquitin ligases distinct from APC/CCdh1. See [36] for C-terminal domain alignment.

### Role of Sororin, Wapl, and Red1 in vegetative growth, sexual development and ascospore viability

To assess the role of Sororin, Wapl, and Red1 during the vegetative phase, we compared the vegetative growth of the ***sororinΔ***, ***waplΔ*,** and ***red1Δ*** null mutants with the wild-type growth rate. To do so, the four strains were inoculated on M2 minimal medium plates and incubated at 25 °C for four days until the plates were fully covered by the mycelium. Three plates were used for each strain and the mycelium growth was measured twice daily. All strains showed similar mycelial phenotypes. However, while the *red1Δ* mutant exhibited a growth rate indistinguishable from that of the wild type, the *sororinΔ* mutant showed only a slight decrease in growth rate (~10%, 2.00 cm/day compared to 2.26 cm/day for WT). In contrast, the *waplΔ* mutant displayed a pronounced and significant reduction in growth rate (~32%, 1.54 cm/day compared with the wild-type rate of 2.26 cm/day) (S3A Fig and S1 Data). These results indicate that Wapl, but not Red1, is required for wild-type vegetative growth, while the small reduction observed in *sororinΔ* does not support a functional requirement for Sororin in this process. We further observed that the *sororin*Δ *waplΔ* double mutant exhibits the same growth rate as the *waplΔ* single mutant, indicating that *waplΔ* is epistatic to *sororinΔ* with respect to vegetative growth phenotype (S3A Fig and S1 Data).

To analyse the transition from the vegetative to the sexual cycle, we monitored the protoperithecia (fruiting bodies) development over 5 days. No differences were observed in the timing or formation of protoperithecia between the null and wild-type strains, suggesting that Sororin, Wapl, and Red1 are not required for the initiation of the sexual cycle. However, clear defects were observed at later stages. Asci development and ascospore formation were assessed by dissecting perithecia after five days of growth. All three mutants exhibited impaired development compared with the wild-type strain, in both ascus differentiation and ascospore morphology (S3B Fig and S1 Data).

In the *sororinΔ* mutant, no asci containing eight wild-type-like ascospores were observed. Instead, 83.9% of the asci aborted before the sporulation step (blue arrow) as indicated by their numerous vacuoles (green arrow). The remaining 16.1% asci contained ascospores, but with abnormal shapes (pink asterisk).

In the *waplΔ* mutant, 70% of asci contained numerous vacuoles (green arrow) and did not form eight mature ascospores, while the remaining 30% of asci exhibited eight ascospores like the wild-type strain (red arrow).

In the *red1Δ* mutant, 41% of asci were either aborted or contained numerous vacuoles (blue and green arrows), while the remaining 59% contained eight wild-type like ascospores (red arrow).

Finally, we examined the ascospore germination of the three null mutants. For this, 80 ascospores were isolated from each strain and deposited on the germination medium. After 24 h at room temperature, the percentage of germinated ascospores was determined. When compared to wild type, the germination rate of the *sororin∆* mutant was strongly reduced (~77%), while absence of Wapl resulted in a moderate reduction (~27%). In contrast, the *red1Δ* mutant showed a wild-type germination rate (S3C Fig and S1 Data).

Taken together, these results demonstrate that Sororin and Wapl are essential for successful sexual reproduction, affecting ascus development, ascospore morphology, and germination efficiency. By contrast, Red1 is dispensable for vegetative growth but still contributes to normal ascus and ascospore development.

Importantly, in all complemented strains, expressing ectopic GFP-tagged proteins in the corresponding null strains, growth rates, ascus development, ascospore morphology and germination rates were indistinguishable from wild type, demonstrating that the GFP-fusion proteins are functional and do fully complement the defects observed in *sororinΔ*, *waplΔ* and *red1Δ* mutants (S3A, S3B, S3C Figs and S1 Data).

### Loading dynamics and localization of Red1, Rad21, Wapl and Sororin during wild-type meiotic prophase I

The localization of the four proteins was examined by single-cell imaging using fully functional GFP fusions: C-terminal GFP fusions for Rad21, Wapl and Sororin and an N-terminal GFP fusion for Red1 (hereafter referred to as GFP-Red1). All fusion proteins were expressed under the control of their native promoters.

**Fig 1:**
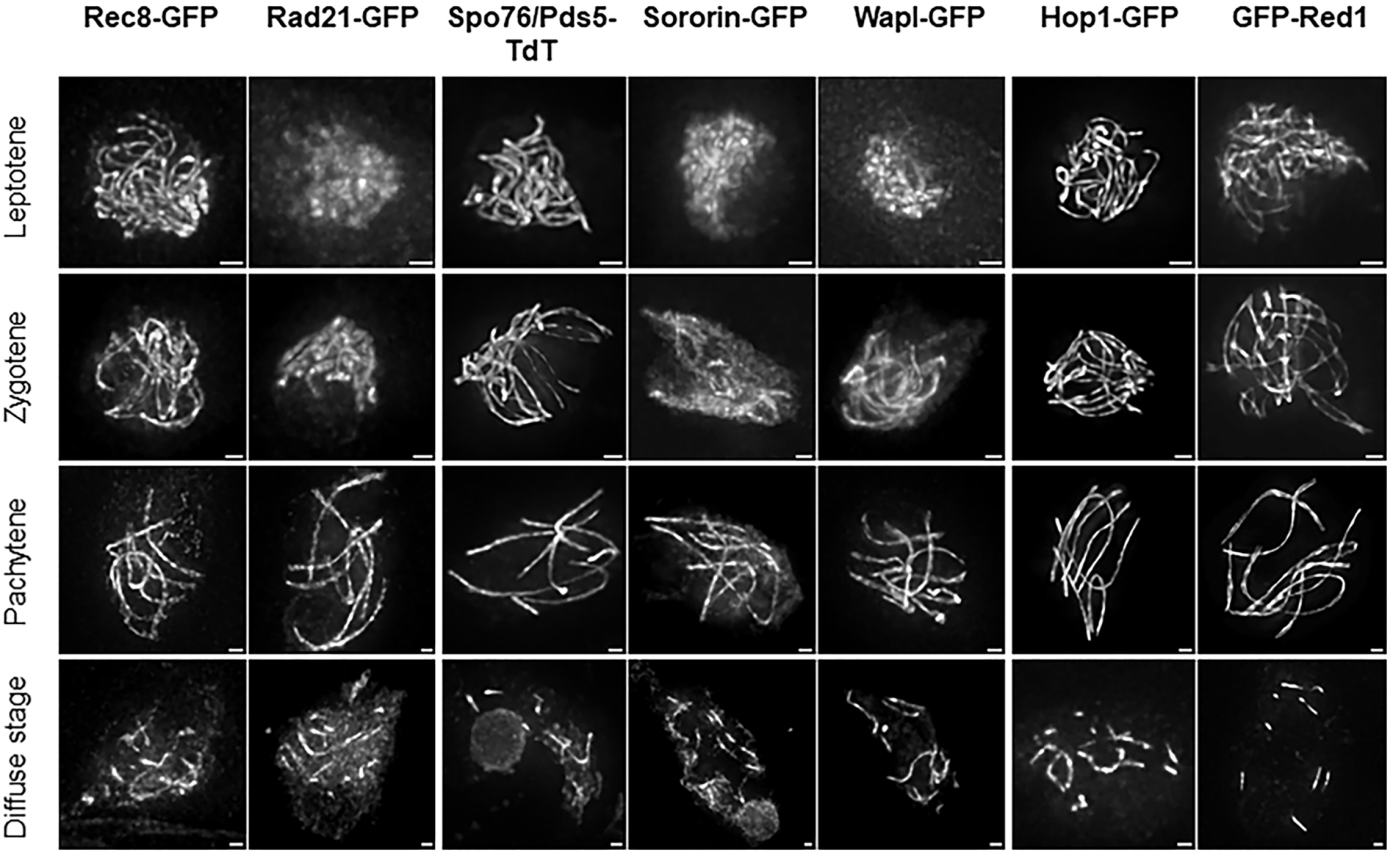
Localization of Rec8, Rad21, Spo76/Pds5, Sororin, Wapl, Hop1 and Red1 during wild-type meiotic prophase I. Single-cell fluorescence imaging of GFP- or TdTomato-tagged proteins shows that all seven proteins localize to chromosome axes from zygotene to late pachytene. Meiotic stages were assigned based on chromatin morphology, synapsis status and ascus size. At leptotene, Rec8, Spo76/Pds5, Hop1 and Red1 appear as continuous lines, whereas Rad21, Wapl and Sororin display diffuse chromatin-associated signals. At pachytene, all seven proteins exhibit a continuous localisation along the entire length of the chromosomes. Their signals progressively decline after pachytene and become restricted to short segments during the post-pachytene diffuse stage. For each strain and for each prophase stage, we analysed a minimum of 20 nuclei n ≥ 20. Scale bars: 1 µm.

To allow a reliable comparison with these newly identified proteins, we first confirmed the previously reported *Sordaria* localization patterns of Rec8-GFP, Hop1-GFP, and Spo76/Pds5-TdTomato in the wild-type strain [12,41,42] (Fig 1).

As shown previously, **Rec8**, **Spo76/Pds5**, and **Hop1** localize along the full length of the chromosome axes from early leptotene on and remain stably associated with the axes until the end of pachytene. Their signals provide robust markers for monitoring homolog recognition during zygotene and homolog synapsis during pachytene. Synapsis corresponds to the assembly of the synaptonemal complex, which aligns homologous chromosomes into close proximity (~100 nm), appearing as a single linear signal under fluorescence microscopy. At the end of pachytene, their signal progressively declines throughout the post-pachytene diffuse stage, and is no longer detectable by diplotene (Fig 1 and S4 Fig).

Consistent with its role as an axial-element protein, **Red1** exhibits the same loading and localization dynamics as Hop1. GFP-Red1 is localized on the axis from very early leptotene on, and runs the full length of the chromosomes as smooth continuous lines until the end of pachytene (Fig 1, right).

In contrast, **Wapl** and **Sororin** are detected on chromatin at early leptotene, and their staining appears as short stretches on the axes at late leptotene. Both signals extend along the entire chromosome axis throughout pachytene (Fig 1).

Interestingly, the two kleisins **Rec8** and **Rad21** display distinct loading patterns at leptotene. Unlike Rec8, which is located on the chromosome axes at early leptotene, Rad21 colocalizes with the chromatin at leptotene but is only detectable on the axes from zygotene onward (Fig 1, left). This localization difference suggests that, in *Sordaria*, Rec8- and Rad21-containing cohesin complexes are sequentially loaded onto DNA during early prophase I. By pachytene, however, their staining patterns converge: both are detected along the full length of the axis and remain detectable as short stretches during the diffuse stage (Fig 1, left bottom). These observations indicate that, in *Sordaria*, both cohesin-complexes populations coexist during meiosis but exhibit distinct loading kinetics in early prophase I.

To address the relevance of Wapl, Red1 and Sororin together with Rad21, Rec8 and Spo76/Pds5 for chromosome-axis structure during meiotic prophase, we isolated and analyzed both single and double null alleles of the corresponding genes to test both their localization and roles in chromosome-axis morphology and synapsis.

### Wapl controls the association of cohesin components and axis proteins with the meiotic chromosome axes and is required for synapsis and chromosome compaction

**Fig 2:**
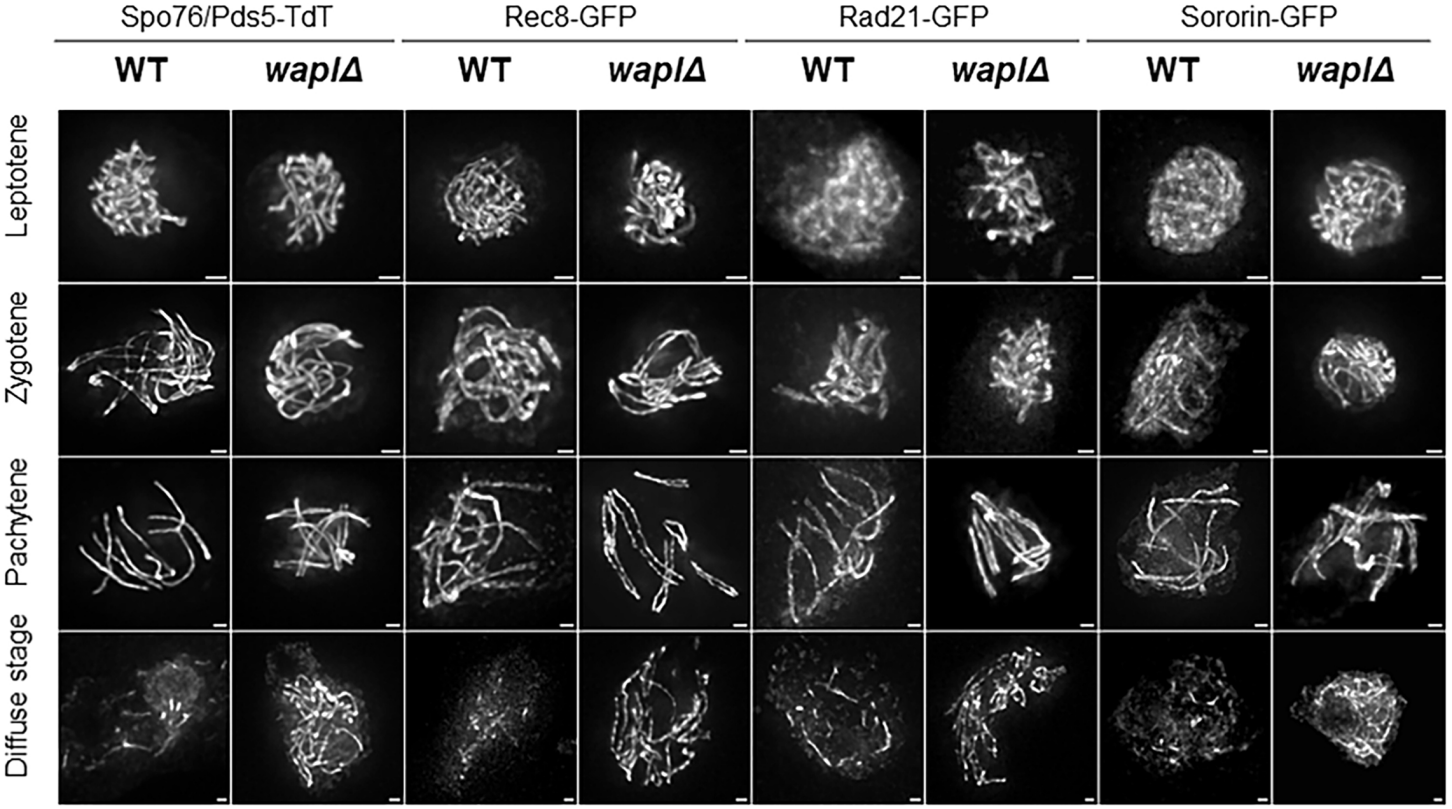
Localization patterns of Spo76/Pds5, Rec8, Rad21, and Sororin in absence of Wapl. Localization patterns of the four cohesin components in wild type (WT) and *wapl∆* from leptotene to the diffuse stage. Note that: **(i)** In WT, Rad21 and Sororin are not detected on chromosome axes at leptotene and appear only from zygotene on, whereas in *wapl∆* they are already associated with the axes at leptotene. **(ii)** At pachytene, Spo76/Pds5, Rec8, Rad21, and Sororin form single continuous lines along each of the seven bivalents because being synapsed at ~100 nm, homologous chromosome axes are indistinguishable in fluorescence microscopy. In contrast, in *wapl∆*, the two homologous axes are visible because of synapsis defects (see details and defects in text). **(iii)** As clear from the magnification bars (identical in WT and mutant), chromosome axes are shorter in *wapl∆* compared to WT (details in text). **(iv)** At the diffuse stage, all four cohesin components persist along chromosome axes in *wapl∆*, whereas in WT they are restricted to short segments. n ≥ 20 nuclei for each strain and for each prophase stage. Scale bars: 1 µm.

To determine the role of Wapl during prophase I, we analyzed the localization of Spo76/Pds5, Rec8, Rad21 and Sororin (Fig 2) as well as of the axis proteins Hop1 and Red1 (S5 Fig) in the *wapl∆* mutant. Like in wild type, all six proteins localize along the homolog axes from leptotene throughout pachytene in the null mutants. However, the absence of Wapl results in the persistent association of these proteins with chromosome axes beyond pachytene, through the diffuse stage, diplotene, and up to anaphase I (Fig 2 and S5 Fig; S4 Fig for diplotene).

This sustained persistent localization highlights the role of *Sordaria* Wapl as a releasing factor that promotes the timely dissociation of cohesin components (Spo76/Pds5, Rec8, Rad21, and Sororin) from meiotic axes, consistent with its conserved function in other organisms (review in [2]). Furthermore, our data reveal that Wapl also negatively regulates the retention of axis proteins Hop1 and Red1, revealing a previously uncharacterized function of Wapl in axis protein dynamics.

Interestingly, in the *waplΔ* mutant, **Rad21** and **Sororin** are already detectable on chromosome axes from leptotene on, in contrast to their later axis localization in wild type. This premature association suggests that Wapl acts not only as a releasing factor at later stages, but also functions earlier during prophase I to prevent stabilization of Rad21 and Sororin on the axes (Fig 2).

We propose that Wapl regulates protein dynamics on meiotic chromosomes at two distinct stages: first, by limiting early axis association of Rad21 and Sororin during leptotene, and later, by facilitating their removal during the diffuse stage. While these activities may reflect a common underlying mechanism, our results do not exclude the possibility that Wapl interferes with initial loading *via* a separate pathway.

Despite proper localization of cohesin components and axis proteins (Red1 and Hop1) throughout prophase I, indicating that, axis assembly *per se* is not impaired in the *waplΔ* mutant, we observed that homologous chromosomes are paired but fail to achieve full synapsis.

To assess this defect, we classified the synaptic status of individual homolog pairs (bivalents) in each nucleus into three categories: (A) complete synapsis (arrow in S6A Fig), (B) partial synapsis (asterisk in S6A Fig), or (C) absence of synapsis (arrowhead in S6A Fig). (*n* = 34 nuclei, 238 bivalents; S6A Fig). Our analysis reveals that only 10,9% of the 238 bivalents display complete synapsis, whereas 89,1% exhibit synapsis defects: 45,8% show partial synapsis and 43,3% show a complete absence of synapsis (S1 Data, values are summarized in Table 1). Notably, we did not observe any nucleus in which all seven bivalents achieved full synapsis. These findings show that Wapl is essential for the establishment of complete homolog synapsis. This synapsis defect is further supported by the localization of the synaptonemal complex (SC) transverse filament protein Sme4/Zip1-GFP. In wild-type nuclei, Sme4/Zip1-GFP forms a continuous linear signal along the full length of each bivalent at pachytene and colocalizes with Spo76/Pds5-TdT (S6B Fig top). By contrast, in *waplΔ* mutants, Sme4/Zip1-GFP signal is frequently discontinuous (arrows in S6B Fig bottom), consistent with incomplete synapsis, and appears continuous only in fully synapsed bivalents.

**Table 1:** Distribution of bivalent synapsis categories in *waplΔ* and *hop1Δ waplΔ* strains.

	No. of bivalents				Percentage of bivalents		
Strain genotype	Fully synapsed	No synapsis	Partial synapsis	Total	Fully synapsed	No synapsis	Partial synapsis
*waplΔ*	26	103	109	238 (n = 34)	10.92	43.28	45.8
*hop1Δ waplΔ*	20	115	40	175 (n = 25)	11.43	65.71	22.86

For each strain, bivalents were scored as fully synapsed, unsynapsed, or partially synapsed. The table reports the number and percentage of bivalents in each category, as well as the total number of bivalents analyzed (with the number of nuclei scored indicated in parentheses).

We further measured chromosome axis length using Spo76/Pds5-TdTomato as a marker in the *waplΔ* mutant. At pachytene, this marker allows accurate tracing of chromosome axes regardless of the synapsis status (S6A Fig for representative tracings). Axis length is significantly reduced in the *waplΔ* mutant compared to wild type (41.74 ± 5 μm, *n* = 34 *vs*. 66.12 ± 7 μm, *n* = 67; Welch’s t test p < 10^-4^; S1 Data). Notably, similar axis lengths are observed in both synapsed and unsynapsed bivalents, indicating that chromosome compaction level is independent of synapsis extent.

Taken together, these findings demonstrate that Wapl regulates both the temporal association of cohesin and axis components as well as the higher-order organization of meiotic chromosomes.

### Sororin protects Spo76/Pds5 and Rec8 from Wapl-mediated release during early prophase I but is not required for Hop1 or Red1 localization

Given that Sororin counteracts Wapl’s cohesin release activity through its association with Spo76/Pds5 in mouse cells [21], we investigated the impact of its absence on chromosome axis structure in *Sordaria*.

**Fig 3:**
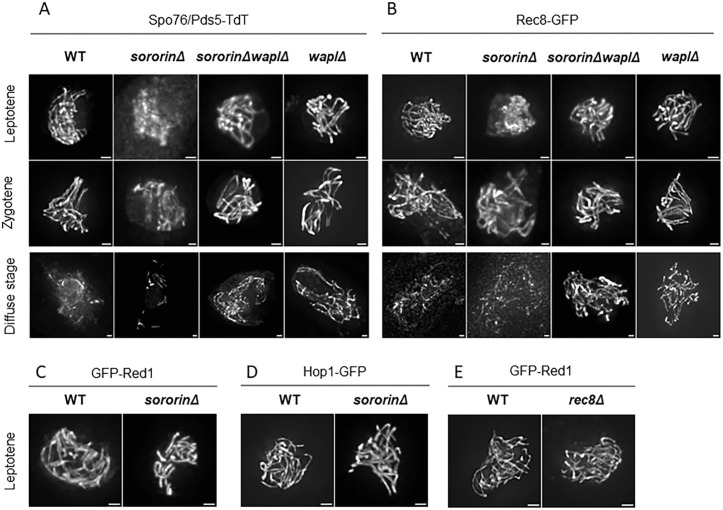
Sororin prevents premature Wapl-mediated release of Spo76/Pds5 and Rec8 but is dispensable for Red1 and Hop1 localization. **(A-B)** Localisation of Spo76/Pds5-TdT and Rec8-GFP in *sororinΔ* single and *sororin*Δ *waplΔ* double mutants compared to their localization in WT and *wapl∆*. In *sororinΔ*, Spo76/Pds5-TdT and Rec8-GFP show only weak chromatin-associated staining at leptotene, and become only visible on axes from zygotene. In contrast, in the *sororin*Δ *waplΔ* double mutant, both proteins are located along chromosome axes from early leptotene on, and persist along axes throughout the diffuse stage, similarly to their location in the *waplΔ* single mutant. **(C-D)** In the absence of Sororin, GFP-Red1 and Hop1-GFP are associated with chromosome axes at leptotene, as in WT. **(E)** GFP-Red1 in *rec8Δ*. Like in WT, Red1 associates with chromosome axes at leptotene in the absence of Rec8*.* n ≥ 20 nuclei for each strain and for each prophase stage. Scale bars: 1 µm.

When compared to wild type, *sororinΔ* exhibits delayed axis localization of **Spo76/Pds5** (Fig 3A) and **Rec8** (Fig 3B): both proteins show weak chromatin-associated staining at leptotene, and become clearly associated with axes only from zygotene onwards (Fig 3A and 3B). These results suggest that Sororin is required for timely recruitment and/or stabilization of Spo76/Pds5 and Rec8 at leptotene, potentially *via* direct or indirect interactions, or by protecting them from premature Wapl-mediated release.

In contrast, in the absence of Sororin, **GFP-Red1** (Fig 3C) and **Hop1-GFP** (Fig 3D) are associated with the axes at leptotene like in wild type. Thus, recruitment of Red1 and Hop1 on the axes does not require Sororin nor the early presence of Spo76/Pds5 and Rec8. This finding differs from the observations made in *S. cerevisiae*, where Red1 loading partially depends on Rec8 [48,49]. However, it is consistent with data from mouse, where it has been shown that SYCP2 (Red1 homolog) localizes on axes in the absence of Rec8 [50,51]. Localization of GFP-Red1 along chromosome axes in the *rec8∆* mutant (Fig 3E) strengthens our conclusions that, in *Sordaria,* Sororin is not required for Red1 loading on axes at leptotene.

Finally, analysis of double mutants shows that the absence of Wapl in the *sororinΔ* mutant restores **Spo76/Pds5** and **Rec8** axis association. Both proteins are again detected along the chromosome axes from early leptotene on in the *sororin*Δ *waplΔ* double mutant (Fig 3A and 3B), indicating that Sororin protects these components from Wapl-dependent release during early prophase I.

Consistent with Wapl’s role as a cohesin releasing factor, Spo76/Pds5 and Rec8 axis staining persists during the diffuse stage in the *sororin*Δ *waplΔ* double mutant, like in the *waplΔ* single mutant (Fig 3A and 3B), demonstrating that *waplΔ* is epistatic to *sororinΔ* with respect to Spo76/Pds5 and Rec8 localization.

Together, these results indicate that Sororin safeguards Spo76/Pds5 and Rec8 from premature Wapl-mediated release during early prophase I, whereas the localization of axis proteins Red1 and Hop1 is Sororin-independent, highlighting a specific protective role of Sororin in cohesin dynamics.

### Hop1 protects Spo76/Pds5 from Wapl-mediated release during pachytene

**Fig 4:**
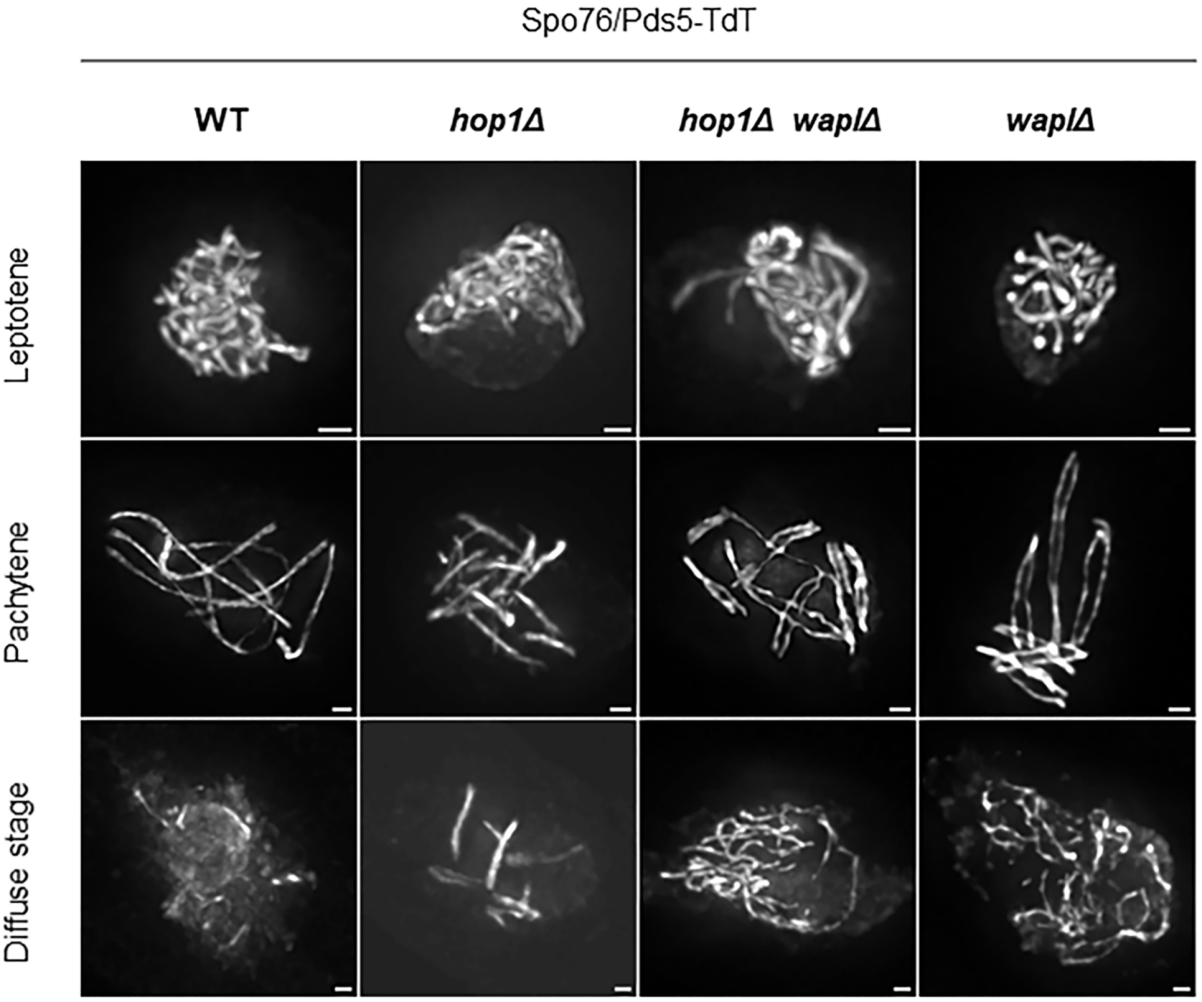
Localization of Spo76/Pds5 in *hop1Δ* single and *hop1Δ* *waplΔ* double mutants compared with its localization in WT and *wapl∆.* In *hop1Δ*, although Spo76/Pds5-TdT initially associates with chromosome axes at leptotene, its signal is specifically lost from certain regions at pachytene, thus forming 13-14 short segments [41]. In contrast, in the *hop1*Δ *waplΔ* double mutant, Spo76/Pds5-TdT is now visible along the entire chromosome axes at pachytene and persists throughout the diffuse stage like in the *waplΔ* single mutant. n ≥ 20 nuclei for each strain and for each prophase stage. Scale bars: 1 µm.

Previous work has shown that Hop1 is required to maintain Spo76/Pds5 along chromosome axes at pachytene: although present along the axes during leptotene and zygotene, Spo76/Pds5 signal is lost in *hop1Δ*, specifically in regions that lack a synaptonemal complex ( [41] and Fig 4). This indicates that Hop1 is not required for Spo76/Pds5 loading on the chromosome axes at early stages but is essential for its local stabilization on chromosome axes during pachytene. We now show that Red1 displays a similar local destabilization pattern along homologs in *hop1Δ* during pachytene (S7A Fig), even though its initial loading onto chromosome axes is independent of Hop1 (S7B Fig).

To test whether this destabilization is mediated by Wapl, we examined Spo76/Pds5 localization in the *hop1*Δ *waplΔ* double mutant. In this background, Spo76/Pds5-TdT is visible along the entire length of the homologous chromosomes at pachytene (Fig 4). This result strongly suggests that Wapl is responsible for the partial loss of Spo76/Pds5 in the *hop1Δ* mutant and that Hop1 protects Spo76/Pds5 from Wapl-mediated removal at pachytene. Strikingly, GFP-Red1 localization is also rescued in the double mutant and is now located along the entire chromosomal axes (S7B Fig). The concomitant restoration of Red1 and Spo76/Pds5 in the *hop1*Δ *waplΔ* mutant suggests a functional interdependence between these proteins. This interdependence could reflect a scenario in which stabilization of Spo76/Pds5 indirectly promotes Red1 association.

We next assessed homolog synapsis in the *hop1Δ waplΔ* double mutant. The synapsis defects are similar to those observed in the single *waplΔ* mutant (Fig 4 and S7B Fig). Among the 175 bivalents analyzed (25 nuclei), only 11,5% achieve complete synapsis, whereas 88,5% exhibit synapsis defects: 23% show partial synapsis and 66% show a complete absence of synapsis (S1 Data, values are summarized in Table 1). Notably, the proportion of unsynapsed bivalents is significantly increased in the *hop1Δ waplΔ* double mutant compared to *waplΔ* (66% *vs* 43%, respectively, 2way Anova p < 10^-4^), suggesting that Hop1 contributes to synapsis in the absence of Wapl, although whether this reflects a direct role in synapsis in this context or an indirect effect through cohesin dynamics remains to be determined.

### Red1 is essential for Hop1 and Spo76/Pds5 axis localization, but is not required for axis association of Rad21 and Rec8

As both Sororin and Hop1 contribute to the axis localization of Spo76/Pds5 in a stage-specific manner, we next asked whether Red1, a known partner of Hop1 in budding yeast [49,52], is required for the localization of cohesin components on meiotic chromosome axes.

**Fig 5:**
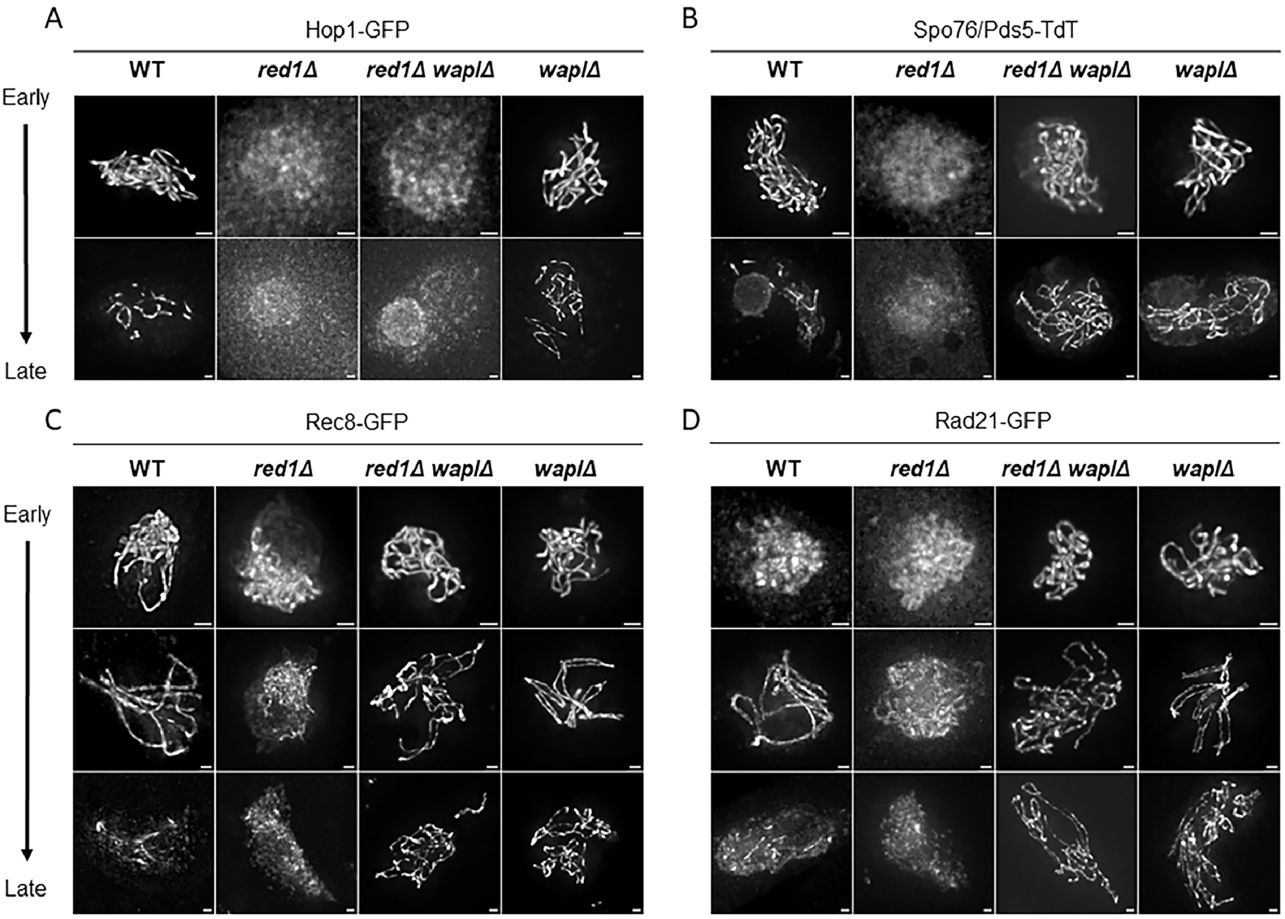
Hop1, Spo76/Pds5, Rec8 and Rad21 localizations in *red1∆* and *red1∆* *wapl∆.* **(A-B)** Hop1-GFP and Spo76/Pds5-TdT localization in *red1Δ* single and *red1*Δ *waplΔ* double mutants compared to their localization in WT and *wapl∆* single mutant. Because reliable axis markers are lacking and chromatin morphology is severely disrupted in *red1Δ*, nuclei were staged as early, mid and late prophase based on ascus size. WT nuclei of comparable ascus size were used as reference, with early corresponding to leptotene, mid-prophase to pachytene, and late prophase to the diffuse stage. In *red1Δ* both proteins fail to associate with axes appearing only as diffuse chromatin-associated signal throughout prophase. In the *red1*Δ *waplΔ* double mutant, Hop1-GFP (A) remains absent on axes while Spo76/Pds5 (B) is located along chromosome axes from early to late prophase. **(C-D)** Rec8-GFP and Rad21-GFP axis localization in *red1Δ* single and *red1*Δ *waplΔ* double mutants compared to their localization in WT and *wapl∆* single mutant. **(C)** In the single *red1Δ* mutant, Rec8 still associates with axes throughout prophase I but is observed only as short stretches or foci, contrasting with its continuous axis staining seen in WT. In contrast, in the *red1*Δ *waplΔ* double mutant, Rec8 forms now continuous lines along axes from early to late prophase, like in the *waplΔ* single mutant. **(D)** At early prophase, Rad21 is chromatin-associated in *red1Δ* like in WT. At mid prophase, Rad21 still associates with axes in *red1Δ* but, unlike in WT, its staining is never observed along the full chromosome axis length. Absence of Wapl in the *red1Δ* background restores premature and continuous Rad21 axis association from early prophase on, with staining persisting through late prophase as in *waplΔ*. n ≥ 20 nuclei for each strain and for each prophase stage. Scale bars: 1 µm.

We first observed that Red1 is essential for the axis association of both **Hop1** (Fig 5A) and **Spo76/Pds5** (Fig 5B) during prophase I. In the *red1Δ* mutant*,* both proteins display diffuse nuclear or chromatin-associated signals and never form linear axis-associated structures (Fig 5A and 5B). Absence of these axis markers prevents the visualization and analysis of pairing and synapsis in the *red1Δ* mutant*.* We, therefore, staged nuclei as early, mid, and late prophase, based on their ascus sizes.

Absence of Hop1-GFP in the *red1Δ* mutant shows that, in *Sordaria* like in budding yeast (e.g., [53]), the localization of Hop1 on axes requires the presence of Red1 (Fig 5A), whereas Red1 can associate with axes independently of Hop1 (S7B Fig).

In contrast, in absence of Red1, **Rec8-GFP** and **Rad21-GFP** exhibit foci and short axis-like stretches but their staining does never extend along the full-length of the chromosomes (Fig 5C and 5D). Thus, both kleisins can associate with chromosome axes independently of Red1, and consequently of Hop1 and Spo76/Pds5. However, Rec8 and Rad21 localization is clearly impaired compared to wild type, where both proteins form continuous axis-associated signals. These results suggest that Red1, Hop1, and/or Spo76/Pds5 promote the stable axis association of Rec8 and Rad21 along chromosome axes during prophase I.

### Red1 protects Spo76/Pds5 from Wapl release and ensures proper chromosome architecture

Given the role of Wapl in promoting the release of Spo76/Pds5, Rec8 and Rad21 from chromosomes in both *sororinΔ* and *hop1Δ* mutants (see above), we next asked whether the loss of Wapl could restore axis association of these proteins in a *red1Δ* mutant background. To address this, we examined their localization in the *red1*Δ *waplΔ* double mutant.

First, **Hop1-GFP** remains undetectable on chromosome axes in the *red1Δ waplΔ* double mutant (Fig 5A), showing that Hop1 axis association strictly requires Red1 and does not involve the Wapl activity. Surprisingly, although **Spo76/Pds5** is completely absent from chromosome axes in the *red1Δ* single mutant, in the *red1Δ waplΔ* double mutant, Spo76/Pds5 becomes clearly detectable along the chromosome axes from early to late prophase I (Fig 5B). This finding strongly indicates that the loss of Spo76/Pds5 on axes in the absence of Red1 is independent of Hop1 and is strongly, if not exclusively, due to the Wapl release activity.

We next examined the localization of the kleisins Rec8-GFP and Rad21-GFP in the *red1*Δ *waplΔ* mutant. Unlike their predominantly punctate or discontinuous staining observed in the *red1Δ* mutant, both proteins now form continuous lines along chromosome axes in the double mutant (Fig 5C and 5D, respectively). Notably, Rad21-GFP is already present on axes at early prophase I, as in the w*aplΔ* mutant (Fig 5D, Top panel), confirming that Wapl prevents its premature association with chromosome axes. Furthermore, Spo76/Pds5, Rec8 and Rad21 staining persists during late prophase in the *red1*Δ *waplΔ* mutant, similar to their staining pattern in the *waplΔ* mutant (Fig 5B, 5C and 5D, Bottom panel). These observations are in line with the Wapl releasing activity and show that *waplΔ* is epistatic to *red1Δ* with respect to Spo76/Pds5 localization.

However, despite the presence of Spo76/Pds5, Rec8, and Rad21 on axes in the *red1*Δ *waplΔ* mutant, chromosome organization remains severely defective. Throughout prophase I, chromatin appears entangled, with no discernible individual chromosomes, even at mid-prophase stages (defined by ascus size), when the 7 bivalents are clearly distinguishable in wild type and *waplΔ* (S8 Fig). These structural defects closely resemble those observed in the *red1Δ* single mutant (S8 Fig), indicating that *red1Δ* is epistatic to *waplΔ* regarding chromatin organization.

Thus, although cohesin complexes are retained on the axes in the *red1*Δ *waplΔ* mutant context, their presence alone is insufficient either to restore wild-type axis architecture or to drive the enhanced chromatin compaction characteristic of *waplΔ*. This discrepancy could reflect two non-exclusive scenarios: (i) cohesins are recruited but remain non-functional in the absence of Red1, or (ii) cohesins are functional, but Red1 plays an upstream architectural role indispensable for meiotic chromatin organization and progression.

Our data reveal, thus, a dual genetic relationship: while *waplΔ* restores axis association of Spo76/Pds5, Rec8, and Rad21 in the absence of Red1, *red1Δ* remains epistatic to *waplΔ* regarding the chromatin organization. This highlights the fact that the presence of cohesins on axes is not sufficient to ensure normal axis structures.

Taken together, these results indicate that in the absence of Red1, Wapl activity compromises the stable association of Rec8 and Rad21 with chromosome axes, either directly or indirectly through the loss of Spo76/Pds5. In the latter scenario, Spo76/Pds5 is a primary target of Wapl, and its stabilization on the axes promotes kleisin retention.

### Rec8 and Rad21 stability relies on Spo76/Pds5, independently of direct Wapl targeting

**Fig 6:**
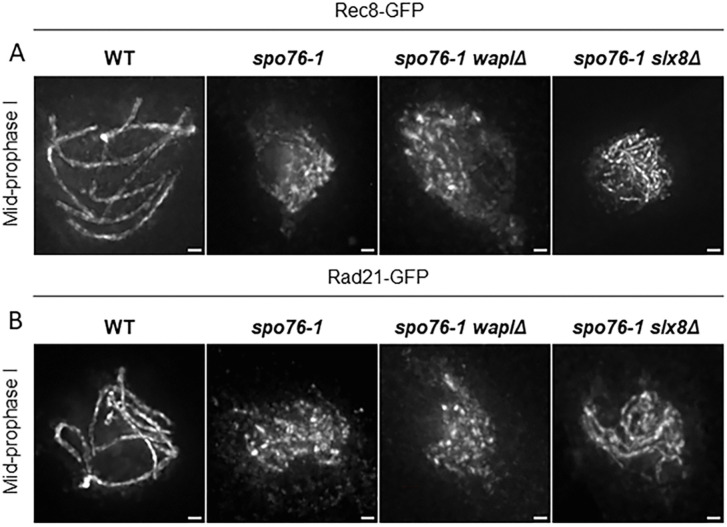
Slx8, but not Wapl, destabilizes Rec8 and Rad21 in the *spo76-1* mutant. **(A-B)** Rec8-GFP (A) and Rad21-GFP (B) in *spo76-1* single, *spo76-1 waplΔ*, and *spo76-1 slx8Δ* double mutants at mid-prophase. In *spo76-1,* like in *red1Δ* (above), chromatin is diffuse and axis markers do not allow reliable staging. Therefore, nuclei were staged based on ascus size, using WT nuclei of comparable size as reference. In the *spo76-1* mutant, Rec8 and Rad21 still associate with chromosome axes but appear as short stretches or discrete foci. Absence of Wapl in the *spo76-1* background does not restore continuous localization of the two kleisins, indicating that Wapl does not act directly on Rec8 or Rad21. Deletion of *SLX8* in the *spo76-1* mutant markedly improves Rec8 and Rad21 axis staining, resulting in sharper and more continuous signals along chromosome axes (n ≥ 20 nuclei). These results indicate that, in the absence of Spo76/Pds5 on the axes, both kleisins are destabilized *via* a Slx8-dependent SUMO-ubiquitin-proteasome pathway. Scale bars: 1 µm.

To assess whether Wapl directly targets kleisins, we analyzed Rec8-GFP and Rad21-GFP localization in the *spo76–1* mutant, which expresses an hypomorphic allele of the essential *SPO76/PDS5* gene in *Sordaria*. In this mutant, **Spo76–1-GFP** localizes to chromatin and forms only discrete foci during prophase I but is never observed along the chromosome axes (S9 Fig). In the *spo76–1* mutant, both Rec8 and Rad21 associate with chromosome axes and exhibit short axis-like stretches and foci during prophase I (Fig 6A and 6B, respectively). This discontinuous staining pattern contrasts with their continuous axis staining in wild type, indicating that Spo76/Pds5 is required for the stabilization of both kleisins on the chromosome axes.

Strikingly, deletion of *WAPL*, in *spo76–1*, failed to restore continuous axis staining of Rec8 or Rad21: both kleisins retained their discontinuous pattern observed in the *spo76–1* single mutant (Fig 6A and 6B, respectively). These findings indicate that: (i) Wapl does not act directly on kleisins, (ii) Spo76/Pds5 protects kleisins from destabilization, and (iii) kleisins are targeted by a distinct pathway in the absence of Spo76/Pds5 from the axes.

### Rec8 and Rad21 are destabilized by the SUMO-targeted ubiquitin ligase Slx8 in the absence of Spo76/Pds5 axis localization

Having established that the destabilization of both kleisins is independent of Wapl activity in the *spo76–1* mutant, we next investigated whether this instability could result from the proteasome-mediated degradation.

In *S. cerevisiae*, the mitotic kleisin Scc1/Rad21 is targeted for degradation in the absence of Spo76/Pds5 *via* the ubiquitin-proteasome system. This process is mediated by the SUMO-targeted ubiquitin ligase (STUbL) complex Slx5-Slx8, which recognizes polySUMOylated cohesin subunits and promotes their ubiquitin-dependent proteolysis [26].

To assess whether this pathway contributes to kleisin destabilization during meiosis in the *spo76–1* mutant, we identified the *Sordaria* homolog of ***SLX8*** (see Methods), deleted the corresponding gene, and examined Rec8 and Rad21 localization along chromosome axes in the absence of Slx8.

Notably, deletion of *SLX8* in the *spo76–1* mutant, leads to a marked improvement in the axial staining of both kleisins, which appear sharper, more continuous, and consistently maintained during prophase I (Fig 6A and 6B).

This improvement of axis association indicates that, in *Sordaria*, Rad21 is targeted during meiosis *via* a Slx8-dependent SUMO-ubiquitin-proteasome pathway, as previously established for mitosis in budding yeast. Importantly, our results show that this regulatory mechanism also applies to the meiotic-specific kleisin Rec8, revealing that Rec8 is similarly susceptible to STUbL-mediated turnover when Spo76/Pds5 is absent from the chromosome axes.

Together, these findings demonstrate that both mitotic and meiotic kleisins are subject to SUMO-ubiquitin-mediated degradation, and that Spo76/Pds5 plays a central protective role, shielding these kleisins from proteasome degradation during meiotic prophase I.

However, deletion of *SLX8* alone does not fully restore the wild-type Rad21 and Rec8 axis localization in the *spo76–1* mutant, indicating that additional factors, which remain to be identified, contribute to kleisins destabilization.

## Discussion

This study explores the roles of the newly identified Wapl, Sororin, and Red1 proteins in *Sordaria macrospora*, focusing on the relationship between cohesins and chromosome axis proteins during prophase I. Using single and double mutant phenotypic analyses, we uncovered new functions for these factors and revealed how their interplay ensures cohesin and axis integrity during meiotic prophase I.

### Crosstalk between cohesin components and axis proteins

The meiotic chromosome axis is composed of the structural proteins Red1 and Hop1 together with cohesin complexes including SMC proteins, kleisins, and associated regulators. Our analysis shows that in *Sordaria,* like in budding yeast and mice [49,52,54], Hop1 recruitment to the axes strictly depends on the prior association of Red1 with axes, whereas Red1 axis loading is independent of Hop1.

Communication among cohesin subunits is well established and critical for both the assembly and regulation of the cohesin complex. In *Sordaria*, deletion of *WAPL* results in persistent retention of cohesin components, premature recruitment of Sororin and Rad21, enhanced chromosome compaction, and defective synapsis. These phenotypes parallel the Wapl functions reported in budding yeast, plants, *Caenorhabditis elegans*, and mammals, indicating that its canonical role as a cohesin releasing factor is conserved in *Sordaria* [13,37,55–59]. Interestingly, our results extend the Wapl function beyond its well-established role at late prophase I. We found that Wapl already acts at leptotene, preventing the early recruitment/localization of Sororin and Rad21 on axes. Thus, Wapl exerts a dual control: first, by delaying the association of these components at early prophase I, and later by promoting cohesin release after pachytene. Whether these two activities rely on the same mechanism, limiting stable binding to axes, or whether Wapl interferes with Sororin and Rad21 initial loading through a distinct pathway remains to be determined.

In mammals, Sororin counteracts Wapl and stabilizes cohesin complexes during meiosis [14,21,36]. Consistently, in *Sordaria*, Sororin is required to maintain Spo76/Pds5 and Rec8 on chromosome axes during early prophase I, protecting them from premature Wapl-mediated release. By contrast, Red1 and Hop1 localization is unaffected in the absence of Sororin, indicating that Wapl specifically targets cohesins and not these two axis proteins.

The interplay between axial elements and cohesins is a central question in meiosis. In *S. cerevisiae*, Rec8-containing cohesin complexes are required for proper Red1 loading, while Red1 and Hop1 contribute to axis integrity and recombination control [49,60,61]. In mouse, SYCP2/SYCP3 (Red1 homologs) can localize independently of Rec8 [50]. These studies highlight that dependencies between axis proteins and Rec8 may vary across species, although the molecular basis of this crosstalk remains unclear. Beyond Rec8, little is known about the interactions between other cohesin subunits and axis proteins.

Our results identify a new protective pathway in which Red1 and Hop1 stabilize Spo76/Pds5 against Wapl-mediated release. In the absence of Hop1, Spo76/Pds5 is specifically lost in the subset of chromosomal regions that lack synaptonemal complex [41], but this loss is reversed when Wapl is deleted, indicating that Hop1 normally shields Spo76/Pds5 from Wapl activity. Likewise, Red1 is essential for Spo76/Pds5 axis association throughout prophase I, and again Wapl removal restores Spo76/Pds5 localization in the *red1Δ* mutant. These findings demonstrate that Red1 and Hop1 do not simply serve as structural axis components but counteract Wapl-mediated release, revealing a new layer of regulation in axis-cohesin crosstalk.

**Fig 7:**
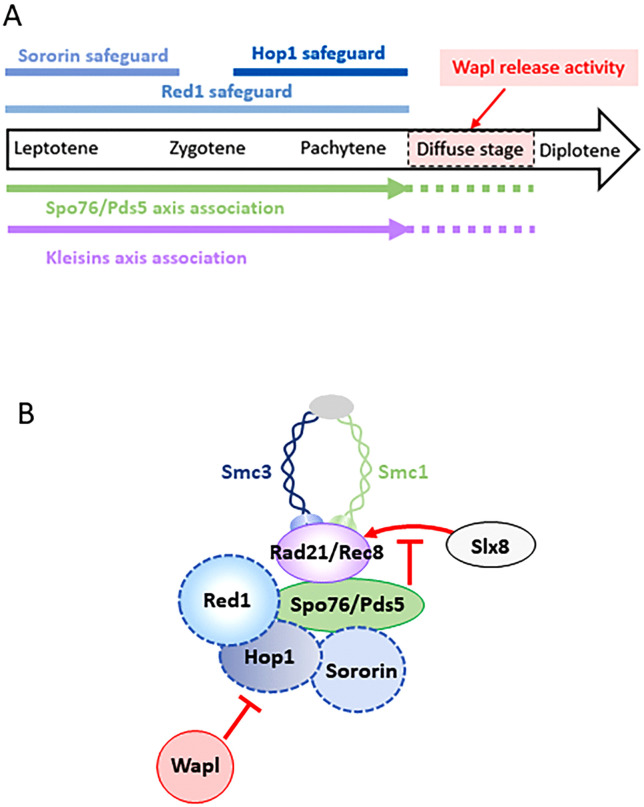
Model for the regulation of cohesin association with meiotic chromosome axes. **(A)** Red1 and Hop1 axis proteins protect the association of cohesin components, Spo76/Pds5 and kleisins, with chromosome axes from Wapl-mediated release. In the absence of Wapl, Spo76/Pds5 and kleisins remain associated with the chromosome axes at the diffuse stage, demonstrating that Wapl ensures the timely cohesin release by the end of pachytene. Red1, Hop1, and Sororin act in a stage-specific manner to protect cohesins: Sororin during leptotene, Hop1 during pachytene, and Red1 throughout prophase **I. (B)** Spo76/Pds5 is a central Wapl target and is stabilized by the combined action of Red1, Hop1, and Sororin. Consistent with this model, these axis proteins become dispensable (for Spo76/Pds5 localization) when Wapl is absent, in agreement with the epistatic interaction of *waplΔ* over *hop1Δ*, *red1Δ*, and *sororinΔ* regarding Spo76/Pds5 axis localization. In turn, Spo76/Pds5 on chromosome axes protects kleisins from ubiquitination and proteasomal degradation *via* the STUbL Slx8 pathway, likely by masking post-translational modification sites.

Moreover, our results indicate that Hop1, Red1, together with Sororin safeguard Spo76/Pds5 in a stage-specific manner: Sororin during early prophase, Hop1 at pachytene, while Red1 provides continuous protection throughout prophase I. This coordinated protection ensures proper cohesin retention and axis integrity (Fig 7A).

Finally, we observed that the persistent retention of cohesins at late pachytene, in the *wapl∆* mutant, correlates with the persistent presence of the axis proteins Red1 and Hop1. Moreover, restoration of full-length Spo76/Pds5 localization in the *hop1*Δ *waplΔ* double mutant is accompanied by a restoration of Red1 on chromosome axes. Since Wapl acts specifically on cohesins, this parallel recovery implies that axis protein maintenance is a downstream consequence of cohesin stability. Conversely, in the *red1*Δ *waplΔ* double mutant, cohesins remain bound to chromosome axes, yet the axis structure is severely disorganized, emphasizing Red1’s central role in meiotic chromosome architecture. These results suggest that Red1 acts as a key structural organizer, essential for proper axis shaping. Alternatively, cohesins may persist in a non-functional state, impaired in loop extrusion or cohesion, indicating that, without Red1, they cannot maintain axis integrity. Clarifying the relative contributions of Red1 and cohesins will be crucial for understanding the mechanisms underlying axis assembly during meiosis.

### Spo76/Pds5 is a central regulator of cohesin’s stability in *Sordaria*

Our data reveal that in *Sordaria*, Spo76/Pds5 functions as a central guardian of cohesin stability during meiotic prophase I. In the *red1Δ* mutant, Wapl efficiently removes Spo76/Pds5 from chromosome axes but is insufficient to fully displace Rec8 and Rad21, suggesting that Spo76/Pds5 is a major target of Wapl activity.

Strikingly, in the *red1*Δ *waplΔ* double mutant, Spo76/Pds5 localization is restored and both kleisins exhibit substantially improved stability along chromosome axes throughout pachytene and beyond. Moreover, in the *spo76–1* hypomorphic mutant, kleisins are destabilized independently of Wapl (in *spo76–1* or in *spo76–1 waplΔ*). These findings indicate that (i) Wapl does not act directly on Rec8 or Rad21 but exerts its activities indirectly *via* Spo76/Pds5 (ii) Spo76/Pds5 functions as a protector of kleisins, whose stability is otherwise compromised in its absence. Deletion of *SLX8* in the *spo76–1* mutant improves the axis association of both kleisins, demonstrating that, Spo76/Pds5 safeguards cohesins from Slx8-dependent SUMO-ubiquitin-proteasome-mediated degradation during meiotic prophase I in *Sordaria*.

Notably, we show here that, like its mitotic counterpart Rad21, the meiotic kleisin Rec8, is subject to STUbL-mediated turnover during meiosis.

The protective role of Spo76/Pds5 may involve multiple mechanisms, especially, it could mask post-translational modification sites on kleisins, such as SUMOylation sites, or recruit deSUMOylating enzymes, thereby preventing recognition by Slx8 and subsequent proteasomal degradation. The partial restoration of kleisin stability in *spo76–1 slx8Δ* suggests that additional, yet unidentified, factors contribute to kleisin destabilization, reflecting a multilayered regulatory network.

### Comparisons with other model systems

Our findings in *Sordaria* highlight both conserved and unique features of cohesin regulation during meiotic prophase. The distinct timing of Rec8 and Rad21 loading, as well as Wapl-dependent control of cohesin retention, are broadly conserved across budding yeast, *C. elegans*, and mammals, indicating a common framework for coordinating axis association and higher-order chromosome organization [37,55,59]. In addition, in *Sordaria*, like in mammals, Sororin antagonizes Wapl by promoting cohesin stabilization [21,36]. In contrast, budding yeast and *C. elegans* lack a canonical Sororin ortholog; in these organisms, regulation of Wapl relies on alternative mechanisms, such as Smc3 acetylation and axis-associated proteins [21–23]. Furthermore, the recruitment of Red1 and Hop1 to chromosome axes in the absence of Sororin, Spo76/Pds5, or Rec8 parallels the behavior of mammalian SYCP2/SYCP3 (Red1 orthologs), but contrasts with budding yeast, where Sororin is absent and Red1 loading is partially Rec8-dependent [44,59,60].

At the same time, *Sordaria* exhibits several distinct features: (i) a stage-specific, coordinated protection of cohesins by Sororin, Hop1, and Red1, (ii) a central role for Spo76/Pds5 as a mediator and major target of Wapl activity, and (iii) Spo76/Pds5 dependent safeguarding of kleisins from SUMO-ubiquitin-dependent proteasome degradation during meiosis. These comparisons emphasize that while the core principles of Wapl regulation and cohesin dynamics are widely conserved, *Sordaria* provides an intermediate and informative model for dissecting both conserved and lineage-specific strategies of cohesin regulation and axis organization during meiosis.

### Concluding remarks

In summary, Sororin, together with the axis proteins Red1 and Hop1, protects Spo76/Pds5 from the Wapl-mediated release. In turn, the presence of Spo76/Pds5 on chromosome axes safeguards kleisins from proteasomal degradation, at least in part *via* the Slx8-dependent STUbL pathway (Fig 7B). This multilayered protection is essential for maintaining cohesin integrity and axis architecture throughout meiotic prophase I.

Given that mutants lacking cohesin and/or axis proteins mostly exhibit also DNA double-strand breaks (DSBs) defects (review in [2]), it remains possible that some of the severe phenotypes observed, particularly in the *red1Δ* mutant, reflect altered DSB metabolism in addition to cohesin defects. Future studies will be essential to disentangle the contributions of recombination, axis assembly, and cohesin regulation, and to define how these pathways converge to ensure faithful axis structure during meiotic progression.

## Methods

### Homolog identification, alignments, phylogeny and models

Homologs were identified by iterative PSI-BLAST searches at NCBI (https://blast.ncbi.nlm.nih.gov/Blast.cgi) or by ProtBLAST/PSI-BLAST and HHMER searches at MPI (https://toolkit.tuebingen.mpg.de) as well as by Foldseek searches (https://search.foldseek.com/search). Multiple sequence alignments (MSAs) generated by MAFFT 7.0 (http://mafft.cbrc.jp/alignment/server), with the auto mode and default parameters, were used as inputs at MPI PSI-BLAST or HHMER websites. Foldseek analyses were done with 3D models from the AlphaFold protein structure database ((https://alphafold.ebi.ac.uk/). Protein modeling was done with the AlphFold3 (AF3) server (https://alphafoldserver.com/). The predicted template modeling (pTM) score and the interface predicted template modeling (ipTM) score were provided by the AF3 website. Protein-protein interaction confidence was evaluated with PIZSA (https://cospi.iiserpune.ac.in/pizsa/). Atomic structures and pLDDT values were visualized in Chimera X (https://www.cgl.ucsf.edu/chimera/).

***SLX8***. *Sordaria* Slx8 (SMAC_06687) is a 418 amino acids protein that displays the two characteristics shared by the STUbL family proteins: one SUMO Interacting Motif (SIM) (VVDL) located at 309–312aa, and a RING-type E3 ubiquitin ligase domain located closer to its C-terminus (339–379aa). The RING domains were identified by SMART (http://smart.embl-heidelberg.de/) or Uniprot (https://www.uniprot.org/) and the putative SIMs were predicted using Jassa (http://www.jassa.fr) and GPS-SUMO (https://sumo.biocuckoo.cn).

### Deletions of the *RED1*, *SORORIN*, *WAPL* and *SLX8* genes

The null *waplΔ*, *red1Δ*, *sororinΔ* and *slx8Δ* mutants were obtained by replacement of their respective entire open-reading frame by the *Escherichia coli hph* gene, conferring hygromycin resistance. The 5’ and 3’ flanking sequences were amplified (S1 Data for primers). Using the Gibson assembly method (NEBuilder HiFi DNA Assembly mix; New England Biolab), both fragments were assembled with pBC-Hygro plasmid, previously cleaved by *XbaI*. This three-partner assembly yielded a deletion plasmid containing a complete deletion of the CDS for each gene. Each plasmid was linearized at the unique XbaI site to generate homologous recombination ends and used to transform a *ku70Δ* mutant strain, known to increase the frequency of homologous integration events. Transformants carrying a null allele were selected for their hygromycin resistance, and the presence of the deleted allele was confirmed through PCR analysis and DNA sequencing. Further crosses with a wild-type *KU70* strain eliminated the *ku70Δ* allele.

### Construction of fluorescent GFP-tagged fusion proteins

The GFP coding sequence (p-EGFP-1, Clontech) was fused immediately after the last C-terminal amino acid predicted from the *WAPL* and *SORORIN* open reading frames and directly after the initiating methionine codon of the *RED1* ORF. For each gene, the GFP fusion coding sequence (CDS) is under the control of its respective promoter. For the GFP-Red1 and Sororin-GFP fusions, a helix-forming linker (LAEAAAKEAAAKEAAAKAAA; Arai et al., 2001) was inserted between GFP and the target protein. In the Wapl-GFP fusion, the linker contains multiple glycine residues (GSGGGGSGGGGG). All constructs were generated using the Gibson assembly method (NEBuilder HiFi DNA Assembly Mix; New England Biolabs). For the Wapl-GFP fusion, the WAPL 5’ flanking region and CDS (excluding the stop codon) were amplified (S1 Data for primers) and assembled with pJET-GSGGGGSGGGGG-GFP, previously digested with BamHI and XhoI. For the Sororin-GFP fusion, the same approach was used: the SORORIN 5’ flanking region and CDS (excluding the stop codon) were amplified and assembled with pBC-LAEAAAKEAAAKEAAAKAAA-GFP, which had been digested with *Sal*I. In both cases, the restriction sites are located upstream of the GFP CDS. The GFP-LAEAAAKEAAAKEAAAKAAA-RED1 allele was generated through a two-step Gibson assembly into the pBC-GFP-LAEAAAKEAAAKEAAAKAAA plasmid (pBC-SK (+), which contains the GFP-helix-forming linker coding sequence inserted at the EcoRV site). First, the 5’ promoter region of *RED1*, amplified by PCR (S1 Data for primers), was inserted upstream of the GFP CDS. In the second step, the *RED1* CDS (including the stop codon) and the 3’ flanking region containing the terminator sequence were inserted downstream, in-frame with the GFP-LAEAAAKEAAAKEAAAKAAA CDS (S1 Data for primers used to amplify this fragment).

After validation by sequencing, GFP-tagged versions of the three genes were ectopically integrated into the WT strain by co-transformation with a plasmid carrying the hygromycin resistance cassette. All resulting strains showed wild-type sporulation.

### Complementation experiments with GFP-tagged proteins

To perform complementation experiments, the Wapl-GFP, GFP-Red1 and Sororin-GFP genes were introduced into the corresponding null mutant strains (*waplΔ*, *red1Δ* and *sororin∆* strains, respectively) by genetic crosses. For each cross, the offspring were genotyped to select *waplΔ* Wapl-GFP, *red1Δ* GFP-Red1 and *sororin∆* Sororin-GFP strains.

### Assessment of vegetative and sexual phenotypes

To evaluate vegetative growth, strains were inoculated onto M2 minimal medium plates and incubated for four days at 25°C until the plates were fully covered by the mycelium. Three plates were used for each strain and the mycelium growth was monitored twice daily by measuring the growth front. The development of protoperithecia and mature perithecia was monitored during ten days. Asci development and ascospore formation were assessed by dissecting perithecia after five days of growth. Perithecia were cracked open using forceps, and ascus bouquets were mounted between slide and coverslip for microscopic observation. Ten ascus bouquets, corresponding to the production of 10 perithecia, were imaged per strain, and the numbers of normal asci (containing eight black ascospores) and abnormal asci were determined. To assess germination efficiency, 80 ascospores were isolated from perithecia and inoculated on germination medium (15g/l BactoAgar, 15g/l Bacto Peptone, 0,06 M Ammonium Acetate). Germination was scored after 24 h at room temperature.

### Cytology

GFP, Tomato-red (TdT) and DAPI (0.5g/ml) signals were observed after fixation in 4% paraformaldehyde. Images were acquired using a Leica DM6 B widefield microscope equipped with a K5 monochrome camera and the THUNDER imager system (Leica Microsystems Inc. Wetzlar, Germany). Thunder computational clearing, which uses Leica Microsystems’ proprietary algorithms was used to enhance image contrast and resolution. The public domain software ImageJ (http://rsb.info.nih.gov/ij) was used for images processing. We used the line tracing tool of the Image J software to measure the axis lengths.

## Supporting information

S1 Fig Red1: schematic domain organization, AlphaFold3 modeling of predicted structural interactions. (A) Domain organization of the *Sordaria* Red1 homolog. The predicted Red1 coiled-coil (CC; aa 583–769), Hop1-interacting region (aa 1–30) as well as the Mek1-interacting domain (aa 170–191) are indicated. Multiple sequence alignment of the Red1 N-terminal region from Sordariales is shown, with amino acid conservation represented using the Clustal color scheme. Hop1- and Mek1-interacting domains are boxed. (B) Schematic representation of Red1 interaction with Hop1, Mek1, Rec114 and Zip4. (C-G) AF3 structural models of predicted Red1 interactions. Ribbon representations are shown with per-residue confidence indicated by pLDDT values (0–50: low confidence, red; 50–70: orange; 70–90: yellow; > 90: high confidence, blue). Predicted template modeling (pTM) and interface pTM (ipTM) scores are reported, together with interaction Z-scores (values >1.5 suggest stable interactions). Expected position error (PAE) plots are shown on the right panels. (C) AF3 structural model of predicted Red1-Hop1 interaction using Red1(aa 1–37) and Hop1 (aa 1–235). (D) AF3 structural modeling of predicted Red1-Mek1 interaction using Red1 (aa 166–244) and Mek1 (full length). (E) AF3 structural modeling of predicted Red1 homodimerization region (aa 568–839). (F) AF3 structural modeling of predicted Red1-Rec114 interaction using Red1 (aa 614–679) and Rec114 (aa 331–401). (G) AF3 structural modeling of predicted Red1-Zip4 interactions using Red1(aa 582–689) and Zip4 (full length).
(TIF)

S2 Fig Wapl: Schematic domain organization and structure. (A) Domain structure and length of *Sordaria* Wapl. The location of the conserved domain is indicated in blue and red bars indicate the location of the putative Pds5-interacting domains [46]. Multiple amino acid sequence alignment of both Pds5-interacting domains is shown, highlighting the N-terminal [M/Φ]xxYG[K/R] motif on the left and the TYxxxR[T/S]ΦL motif on the right. (B, C) AF2-modelled SMAC4_07015/Wapl protein. (B) Rainbow coloring represents the N- to C-terminal gradient as indicated. (C) Model confidence is assessed using the predicted per-residue local distance difference test (pLDDT; left) and the alignment error between all residue pairs (PAE; right). (D) The AlphaFold2-modelled *Sordaria* Wapl domain (left), and the experimentally determined atomic structure of *A. gossypii* Wapl domain (PDB: 3zik, middle) share structural similarities as shown by their superimposition (right). According to the *A. gossypii* Wapl domain structure, the six putative HEAT repeats are respectively colored in cyan, pale blue, blue, yellow, orange, and red [47]. The position of the helical insertion within repeat HEAT3 is indicated in dark grey. The N-terminal and C-terminal region surrounding the HEAT repeats are colored green.
(TIF)

S3 Fig Role of Sororin, Wapl, and Red1 in vegetative growth, ascus development, and ascospore germination. (A) Vegetative growth of wild type (WT), *sororinΔ*, *waplΔ*, and *red1Δ* mutants, as well as of the complemented strains expressing GFP-tagged proteins, was monitored over 96 h on M2 medium. Growth rates of *sororinΔ* and *waplΔ* are reduced (~10% and ~32%, respectively), while *red1Δ* grows like WT. Complementation restores growth to WT levels. (B) Quantification of abnormal asci in each strain (top) and representative images of asci in five-days old perithecia (bottom). In the *sororinΔ* mutant, no asci contain eight wild-type-like ascospores; most asci abort before sporulation, as indicated by their numerous vacuoles (blue and green arrows), while the few remaining asci produce morphologically abnormal ascospores (pink asterisks). In the *waplΔ* mutant, 70% of asci contain numerous vacuoles (green arrow) and do not form eight mature ascospores, while the remaining 30% of asci exhibit eight ascospores like the wild-type strain (red arrow). In the *red1Δ* mutant, 41% of asci are either aborted or contain numerous vacuoles (blue and green arrows), while the remaining 59% contain eight wild-type like ascospores (red arrow). Complemented strains produce normal asci and ascospores. (C) Percentage of germinated ascospores after 24 h. Germination is strongly reduced in *sororinΔ*, moderately reduced in *waplΔ*, and unaffected in *red1Δ*. Complemented strains restored germination to WT levels. The raw data underlying S3Fig. A, B, and C are available in S1 Data.
(TIF)

S4 Fig Localization of Spo76/Pds5, Rec8 and Rad21 in WT and *wapl∆* diplotene nuclei. In WT, none of the three proteins is detectable on chromosome axes, whereas they persist along the axes through *waplΔ* diplotene, indicating that Wapl is required for their timely release from chromosome axes. Right: corresponding DAPI (blue). Scale bar: 1 μm.
(TIF)

S5 Fig Localization of axis proteins Hop1 and Red1 in the absence of Wapl. Comparison with WT shows that, in the *waplΔ* mutant, Hop1 and Red1 exhibit a WT-like localization along chromosome axes from leptotene to pachytene. However, instead of disappearing progressively at the end of pachytene like in WT, their signals persist throughout the diffuse stage in the mutant. n ≥ 20 nuclei for each strain and for each prophase stage. Scale bars: 1 µm.
(TIF)

S6 Fig Axis length and impaired synapsis in the *waplΔ* mutant. (A) Representative *waplΔ* pachytene nucleus with Spo76/Pds5-TdT-labelled chromosome axes. Left: raw image. Middle: colour tracing of individual bivalents allows visualization of the different synapsis defects: full synapsis in the yellow and orange bivalents (arrow on the orange bivalent), partial synapsis in blue and purple bivalents (asterisk on the purple bivalent), or no synapsis (arrowhead in the green bivalent). Right: measurements of the 14 axes indicates that homolog axes have the same length. Total axis length at the bottom (detail in text). (B) Sme4/Zip1-GFP localization in WT (top) and *waplΔ* (bottom). In WT bivalents, Sme4-GFP shows smooth lines from telomere to telomere and colocalizes with Spo76/Pds5-TdT. In contrast, in the *waplΔ* mutant, colocalization with Spo76/Pds5-TdT indicates that Sme4/Zip1-GFP forms only discontinuous stretches (arrows) along the bivalents, indicative of synapsis defects (n ≥ 20 nuclei). Scale bars: 1µm.
(TIF)

S7 Fig During pachytene, loss of Hop1 causes local Red1 destabilization, rescued by absence of *Wapl.* (A) Colocalization of GFP-Red1 and Spo76/Pds5-TdT in the *hop1Δ* mutant at pachytene. The two proteins never exhibit full-length staining along the axes, indicating that they share the same pattern of local destabilization (see merged image of tracing and DAPI, right). DNA regions lacking staining for both proteins are indicated by arrows. (B) GFP-Red1 localization in *hop1Δ* single and *hop1Δ waplΔ* double mutants compared to its WT and *waplΔ* localization. In *hop1Δ*, Red1 associates with axis at leptotene as in WT, but is locally destabilized at pachytene. In the *hop1Δ waplΔ* double mutant, Red1 decorates the full length of the chromosome axes from leptotene to pachytene. The *hop1Δ waplΔ* double mutant exhibits similar synapsis defects as the single *waplΔ* mutant (quantification in text). n ≥ 20 nuclei for each strain and for each prophase stage. Scale bars: 1µm.
(TIF)

S8 Fig Epistatic interaction of *red1Δ* over *waplΔ* for chromatin morphology. DAPI staining of chromatin in WT, *red1Δ*, *red1Δ waplΔ* and *waplΔ* at mid-prophase I (as defined by ascus size). In the *red1Δ* single and *red1Δ waplΔ* double mutants, chromatin appears diffuse and entangled, with no discernible chromosomes, when 7 bivalents are distinguishable in WT and *waplΔ*. This indicates that *red1Δ* is epistatic to *waplΔ* with respect to chromatin organization (n ≥ 20 nuclei). Scale bars: 1 µm.
(TIF)

S9 Fig Localization of Spo76–1-GFP in *spo76–1* mutant. The mutant protein Spo76–1-GFP exhibits mainly chromatin staining during prophase I. Right: corresponding DAPI (n ≥ 20 nuclei). Scale bars: 1 µm.
(TIF)

S1 Data Underlying data for Fig 3 and Table 1. (XLSX)

## References

[1] Bonev B, Cavalli G. Organization and function of the 3D genome. Nat Rev Genet. 2016;17(11):661–78. doi: 10.1038/nrg.2016.112 27739532

[2] Grey C, de Massy B. Chromosome organization in early meiotic prophase. Front Cell Dev Biol. 2021;9:688878. doi: 10.3389/fcell.2021.688878 34150782 PMC8209517

[3] Ur SN, Corbett KD. Architecture and dynamics of meiotic chromosomes. Annu Rev Genet. 2021;55:497–526. doi: 10.1146/annurev-genet-071719-020235 34530636

[4] Hoencamp C, Rowland BD. Genome control by SMC complexes. Nat Rev Mol Cell Biol. 2023;24(9):633–50. doi: 10.1038/s41580-023-00609-8 37231112

[5] Davidson IF, Bauer B, Goetz D, Tang W, Wutz G, Peters J-M. DNA loop extrusion by human cohesin. Science. 2019;366(6471):1338–45. doi: 10.1126/science.aaz3418 31753851

[6] Merkenschlager M, Nora EP. CTCF and cohesin in genome folding and transcriptional gene regulation. Annu Rev Genomics Hum Genet. 2016;17:17–43. doi: 10.1146/annurev-genom-083115-022339 27089971

[7] Peters J-M, Tedeschi A, Schmitz J. The cohesin complex and its roles in chromosome biology. Genes Dev. 2008;22(22):3089–114. doi: 10.1101/gad.1724308 19056890

[8] Nasmyth K, Haering CH. The structure and function of SMC and kleisin complexes. Annu Rev Biochem. 2005;74:595–648. doi: 10.1146/annurev.biochem.74.082803.133219 15952899

[9] Roig MB, Löwe J, Chan K-L, Beckouët F, Metson J, Nasmyth K. Structure and function of cohesin’s Scc3/SA regulatory subunit. FEBS Lett. 2014;588(20):3692–702. doi: 10.1016/j.febslet.2014.08.015 25171859 PMC4175184

[10] Orgil O, Matityahu A, Eng T, Guacci V, Koshland D, Onn I. A conserved domain in the scc3 subunit of cohesin mediates the interaction with both mcd1 and the cohesin loader complex. PLoS Genet. 2015;11(3):e1005036. doi: 10.1371/journal.pgen.1005036 25748820 PMC4352044

[11] Petela NJ, Gligoris TG, Metson J, Lee B-G, Voulgaris M, Hu B, et al. Scc2 Is a Potent Activator of Cohesin’s ATPase that promotes loading by binding Scc1 without Pds5. Mol Cell. 2018;70(6):1134-1148.e7. doi: 10.1016/j.molcel.2018.05.022 29932904 PMC6028919

[12] van Heemst D, James F, Pöggeler S, Berteaux-Lecellier V, Zickler D. Spo76p is a conserved chromosome morphogenesis protein that links the mitotic and meiotic programs. Cell. 1999;98(2):261–71. doi: 10.1016/s0092-8674(00)81020-x 10428037

[13] Kueng S, Hegemann B, Peters BH, Lipp JJ, Schleiffer A, Mechtler K, et al. Wapl controls the dynamic association of cohesin with chromatin. Cell. 2006;127(5):955–67. doi: 10.1016/j.cell.2006.09.040 17113138

[14] Rankin S, Ayad NG, Kirschner MW. Sororin, a substrate of the anaphase-promoting complex, is required for sister chromatid cohesion in vertebrates. Mol Cell. 2005;18(2):185–200. doi: 10.1016/j.molcel.2005.03.017 15837422

[15] Schmitz J, Watrin E, Lénárt P, Mechtler K, Peters J-M. Sororin is required for stable binding of cohesin to chromatin and for sister chromatid cohesion in interphase. Curr Biol. 2007;17(7):630–6. doi: 10.1016/j.cub.2007.02.029 17349791

[16] Srinivasan M, Petela NJ, Scheinost JC, Collier J, Voulgaris M, B Roig M, et al. Scc2 counteracts a Wapl-independent mechanism that releases cohesin from chromosomes during G1. Elife. 2019;8:e44736. doi: 10.7554/eLife.44736 31225797 PMC6588348

[17] Ivanov MP, Ladurner R, Poser I, Beveridge R, Rampler E, Hudecz O, et al. The replicative helicase MCM recruits cohesin acetyltransferase ESCO2 to mediate centromeric sister chromatid cohesion. EMBO J. 2018;37(15):e97150. doi: 10.15252/embj.201797150 29930102 PMC6068434

[18] Ivanov D, Schleiffer A, Eisenhaber F, Mechtler K, Haering CH, Nasmyth K. Eco1 is a novel acetyltransferase that can acetylate proteins involved in cohesion. Curr Biol. 2002;12(4):323–8. doi: 10.1016/s0960-9822(02)00681-4 11864574

[19] Rolef Ben-Shahar T, Heeger S, Lehane C, East P, Flynn H, Skehel M, et al. Eco1-dependent cohesin acetylation during establishment of sister chromatid cohesion. Science. 2008;321(5888):563–6. doi: 10.1126/science.1157774 18653893

[20] Tóth A, Ciosk R, Uhlmann F, Galova M, Schleiffer A, Nasmyth K. Yeast cohesin complex requires a conserved protein, Eco1p(Ctf7), to establish cohesion between sister chromatids during DNA replication. Genes Dev. 1999;13(3):320–33. doi: 10.1101/gad.13.3.320 9990856 PMC316435

[21] Nishiyama T, Ladurner R, Schmitz J, Kreidl E, Schleiffer A, Bhaskara V, et al. Sororin mediates sister chromatid cohesion by antagonizing Wapl. Cell. 2010;143(5):737–49. doi: 10.1016/j.cell.2010.10.031 21111234

[22] Beckouët F, Hu B, Roig MB, Sutani T, Komata M, Uluocak P, et al. An Smc3 acetylation cycle is essential for establishment of sister chromatid cohesion. Mol Cell. 2010;39(5):689–99. doi: 10.1016/j.molcel.2010.08.008 20832721 PMC4766734

[23] Chan K-L, Gligoris T, Upcher W, Kato Y, Shirahige K, Nasmyth K, et al. Pds5 promotes and protects cohesin acetylation. Proc Natl Acad Sci U S A. 2013;110(32):13020–5. doi: 10.1073/pnas.1306900110 23878248 PMC3740900

[24] Borges V, Lehane C, Lopez-Serra L, Flynn H, Skehel M, Rolef Ben-Shahar T, et al. Hos1 deacetylates Smc3 to close the cohesin acetylation cycle. Mol Cell. 2010;39(5):677–88. doi: 10.1016/j.molcel.2010.08.009 20832720

[25] Psakhye I, Branzei D. SMC complexes are guarded by the SUMO protease Ulp2 against SUMO-chain-mediated turnover. Cell Rep. 2021;36(5):109485. doi: 10.1016/j.celrep.2021.109485 34348159

[26] D’Ambrosio LM, Lavoie BD. Pds5 prevents the PolySUMO-dependent separation of sister chromatids. Curr Biol. 2014;24(4):361–71. doi: 10.1016/j.cub.2013.12.038 24485833

[27] Stead K, Aguilar C, Hartman T, Drexel M, Meluh P, Guacci V. Pds5p regulates the maintenance of sister chromatid cohesion and is sumoylated to promote the dissolution of cohesion. J Cell Biol. 2003;163(4):729–41. doi: 10.1083/jcb.200305080 14623866 PMC2173684

[28] Wendt KS, Yoshida K, Itoh T, Bando M, Koch B, Schirghuber E, et al. Cohesin mediates transcriptional insulation by CCCTC-binding factor. Nature. 2008;451(7180):796–801. doi: 10.1038/nature06634 18235444

[29] Bennett MD. The time and duration of meiosis. Philos Trans R Soc Lond B Biol Sci. 1977;277(955):201–26. doi: 10.1098/rstb.1977.0012 16285

[30] Zickler D, Kleckner N. Meiosis: dances between homologs. Annu Rev Genet. 2023;57:1–63. doi: 10.1146/annurev-genet-061323-044915 37788458

[31] Watanabe Y. Modifying sister chromatid cohesion for meiosis. J Cell Sci. 2004;117(Pt 18):4017–23. doi: 10.1242/jcs.01352 15316077

[32] Lin W, Jin H, Liu X, Hampton K, Yu H-G. Scc2 regulates gene expression by recruiting cohesin to the chromosome as a transcriptional activator during yeast meiosis. Mol Biol Cell. 2011;22(12):1985–96. doi: 10.1091/mbc.E10-06-0545 21508318 PMC3113765

[33] Wang H, Xu W, Sun Y, Lian Q, Wang C, Yu C, et al. The cohesin loader SCC2 contains a PHD finger that is required for meiosis in land plants. PLoS Genet. 2020;16(6):e1008849. doi: 10.1371/journal.pgen.1008849 32516352 PMC7304647

[34] Lightfoot J, Testori S, Barroso C, Martinez-Perez E. Loading of meiotic cohesin by SCC-2 is required for early processing of DSBs and for the DNA damage checkpoint. Curr Biol. 2011;21(17):1421–30. doi: 10.1016/j.cub.2011.07.007 21856158

[35] Ding D-Q, Sakurai N, Katou Y, Itoh T, Shirahige K, Haraguchi T, et al. Meiotic cohesins modulate chromosome compaction during meiotic prophase in fission yeast. J Cell Biol. 2006;174(4):499–508. doi: 10.1083/jcb.200605074 16893973 PMC2064256

[36] Prusén Mota I, Galova M, Schleiffer A, Nguyen T-T, Kovacikova I, Farias Saad C, et al. Sororin is an evolutionary conserved antagonist of WAPL. Nat Commun. 2024;15(1):4729. doi: 10.1038/s41467-024-49178-0 38830897 PMC11148194

[37] Challa K, Lee M-S, Shinohara M, Kim KP, Shinohara A. Rad61/Wpl1 (Wapl), a cohesin regulator, controls chromosome compaction during meiosis. Nucleic Acids Res. 2016;44(7):3190–203. doi: 10.1093/nar/gkw034 26825462 PMC4838362

[38] Revenkova E, Eijpe M, Heyting C, Hodges CA, Hunt PA, Liebe B, et al. Cohesin SMC1 beta is required for meiotic chromosome dynamics, sister chromatid cohesion and DNA recombination. Nat Cell Biol. 2004;6(6):555–62. doi: 10.1038/ncb1135 15146193

[39] Kim Y, Rosenberg SC, Kugel CL, Kostow N, Rog O, Davydov V, et al. The chromosome axis controls meiotic events through a hierarchical assembly of HORMA domain proteins. Dev Cell. 2014;31(4):487–502. doi: 10.1016/j.devcel.2014.09.013 25446517 PMC4254552

[40] West AMV, Komives EA, Corbett KD. Conformational dynamics of the Hop1 HORMA domain reveal a common mechanism with the spindle checkpoint protein Mad2. Nucleic Acids Res. 2018;46(1):279–92. doi: 10.1093/nar/gkx1196 29186573 PMC5758881

[41] Dubois E, Boisnard S, Bourbon H-M, Yefsah K, Budin K, Debuchy R, et al. Canonical and noncanonical roles of Hop1 are crucial for meiotic prophase in the fungus Sordaria macrospora. PLoS Biol. 2024;22(7):e3002705. doi: 10.1371/journal.pbio.3002705 38950075 PMC11244814

[42] Storlazzi A, Tesse S, Ruprich-Robert G, Gargano S, Pöggeler S, Kleckner N, et al. Coupling meiotic chromosome axis integrity to recombination. Genes Dev. 2008;22(6):796–809. doi: 10.1101/gad.459308 18347098 PMC2275432

[43] Hollingsworth NM, Ponte L. Genetic interactions between HOP1, RED1 and MEK1 suggest that MEK1 regulates assembly of axial element components during meiosis in the yeast Saccharomyces cerevisiae. Genetics. 1997;147(1):33–42. doi: 10.1093/genetics/147.1.33 9286666 PMC1208117

[44] Panizza S, Mendoza MA, Berlinger M, Huang L, Nicolas A, Shirahige K, et al. Spo11-accessory proteins link double-strand break sites to the chromosome axis in early meiotic recombination. Cell. 2011;146(3):372–83. doi: 10.1016/j.cell.2011.07.003 21816273

[45] De Muyt A, Pyatnitskaya A, Andréani J, Ranjha L, Ramus C, Laureau R, et al. A meiotic XPF-ERCC1-like complex recognizes joint molecule recombination intermediates to promote crossover formation. Genes Dev. 2018;32(3–4):283–96. doi: 10.1101/gad.308510.117 29440262 PMC5859969

[46] Nasmyth KA, Lee B-G, Roig MB, Löwe J. What AlphaFold tells us about cohesin’s retention on and release from chromosomes. Elife. 2023;12:RP88656. doi: 10.7554/eLife.88656 37975572 PMC10656103

[47] Chatterjee A, Zakian S, Hu X-W, Singleton MR. Structural insights into the regulation of cohesion establishment by Wpl1. EMBO J. 2013;32(5):677–87. doi: 10.1038/emboj.2013.16 23395900 PMC3590988

[48] Heldrich J, Milano CR, Markowitz TE, Ur SN, Vale-Silva LA, Corbett KD, et al. Two pathways drive meiotic chromosome axis assembly in Saccharomyces cerevisiae. Nucleic Acids Res. 2022;50(8):4545–56. doi: 10.1093/nar/gkac227 35412621 PMC9071447

[49] Sun X, Huang L, Markowitz TE, Blitzblau HG, Chen D, Klein F, et al. Transcription dynamically patterns the meiotic chromosome-axis interface. Elife. 2015;4:e07424. doi: 10.7554/eLife.07424 26258962 PMC4530585

[50] Agostinho A, Höög C. REC8 density along chromosomes prevents illegitimate synapsis. Cell Cycle. 2016;15(19):2543–4. doi: 10.1080/15384101.2016.1204853 27359070 PMC5053557

[51] Fujiwara Y, Horisawa-Takada Y, Inoue E, Tani N, Shibuya H, Fujimura S, et al. Meiotic cohesins mediate initial loading of HORMAD1 to the chromosomes and coordinate SC formation during meiotic prophase. PLoS Genet. 2020;16(9):e1009048. doi: 10.1371/journal.pgen.1009048 32931493 PMC7518614

[52] de los Santos T, Hollingsworth NM. Red1p, a MEK1-dependent phosphoprotein that physically interacts with Hop1p during meiosis in yeast. J Biol Chem. 1999;274(3):1783–90. doi: 10.1074/jbc.274.3.1783 9880561

[53] Smith AV, Roeder GS. The yeast Red1 protein localizes to the cores of meiotic chromosomes. J Cell Biol. 1997;136(5):957–67. doi: 10.1083/jcb.136.5.957 9060462 PMC2132480

[54] Markowitz TE, Suarez D, Blitzblau HG, Patel NJ, Markhard AL, MacQueen AJ, et al. Reduced dosage of the chromosome axis factor Red1 selectively disrupts the meiotic recombination checkpoint in Saccharomyces cerevisiae. PLoS Genet. 2017;13(7):e1006928. doi: 10.1371/journal.pgen.1006928 28746375 PMC5549997

[55] Crawley O, Barroso C, Testori S, Ferrandiz N, Silva N, Castellano-Pozo M, et al. Cohesin-interacting protein WAPL-1 regulates meiotic chromosome structure and cohesion by antagonizing specific cohesin complexes. Elife. 2016;5:e10851. doi: 10.7554/eLife.10851 26841696 PMC4758955

[56] De K, Sterle L, Krueger L, Yang X, Makaroff CA. Arabidopsis thaliana WAPL is essential for the prophase removal of cohesin during meiosis. PLoS Genet. 2014;10(7):e1004497. doi: 10.1371/journal.pgen.1004497 25033056 PMC4102442

[57] Hong S, Joo JH, Yun H, Kleckner N, Kim KP. Recruitment of Rec8, Pds5 and Rad61/Wapl to meiotic homolog pairing, recombination, axis formation and S-phase. Nucleic Acids Res. 2019;47(22):11691–708. doi: 10.1093/nar/gkz903 31617566 PMC7145551

[58] Ouyang Z, Yu H. Releasing the cohesin ring: a rigid scaffold model for opening the DNA exit gate by Pds5 and Wapl. Bioessays. 2017;39(4):10.1002/bies.201600207. doi: 10.1002/bies.201600207 28220956

[59] Silva MCC, Powell S, Ladstätter S, Gassler J, Stocsits R, Tedeschi A, et al. Wapl releases Scc1-cohesin and regulates chromosome structure and segregation in mouse oocytes. J Cell Biol. 2020;219(4):e201906100. doi: 10.1083/jcb.201906100 32328639 PMC7147110

[60] Klein F, Mahr P, Galova M, Buonomo SB, Michaelis C, Nairz K, et al. A central role for cohesins in sister chromatid cohesion, formation of axial elements, and recombination during yeast meiosis. Cell. 1999;98(1):91–103. doi: 10.1016/S0092-8674(00)80609-1 10412984

[61] Kim KP, Weiner BM, Zhang L, Jordan A, Dekker J, Kleckner N. Sister cohesion and structural axis components mediate homolog bias of meiotic recombination. Cell. 2010;143(6):924–37. doi: 10.1016/j.cell.2010.11.015 21145459 PMC3033573

